# Changes of Ginsenoside Composition in the Creation of Black Ginseng Leaf

**DOI:** 10.3390/molecules25122809

**Published:** 2020-06-18

**Authors:** Wei Chen, Prabhu Balan, David G. Popovich

**Affiliations:** 1 School of Food and Advanced Technology, Massey University, Palmerston North 4442, New Zealand; W.Chen2@massey.ac.nz; 2 Riddet Institute, Massey University, Palmerston North 4442, New Zealand; P.Balan@massey.ac.nz; 3 Alpha-Massey Natural Nutraceutical Research Centre, Massey University, Palmerston North 4442, New Zealand

**Keywords:** *Panax ginseng*, ginseng leaf, ginsenosides, black ginseng, steam-processing

## Abstract

Ginseng is an increasingly popular ingredient in supplements for healthcare products and traditional medicine. Heat-processed ginsengs, such as red ginseng or black ginseng, are regarded as more valuable for medicinal use when compared to white ginseng due to some unique less polar ginsenosides that are produced during heat-treatment. Although ginseng leaf contains abundant ginsenosides, attention has mostly focused on ginseng root; relatively few publications have focused on ginseng leaf. Raw ginseng leaf was steamed nine times to make black ginseng leaf using a process that is similar to that used to produce black ginseng root. Sixteen ginsenosides were analyzed during each steaming while using high-performance liquid chromatography (HPLC). The contents of ginsenosides Rd and Re decreased and the less polar ginsenosides (F2, Rg3, Rk2, Rk3, Rh3, Rh4, and protopanaxatriol) enriched during steam treatment. After nine cycles of steaming, the contents of the less polar ginsenosides F2, Rg3, and Rk2 increased by 12.9-fold, 8.6-fold, and 2.6-fold, respectively. Further, we found that the polar protopanaxadiol (PPD) -type ginsenosides are more likely to be converted from ginsenoside Rg3 to ginsenosides Rk1 and Rg5 via dehydration from Rg3, and from ginsenoside Rh2 to ginsenosides Rk2 and Rh3 through losing an H_2_O molecule than to be completely degraded to the aglycones PPD during the heat process. This study suggests that ginseng leaves can be used to produce less polar ginsenosides through heat processes, such as steaming.

## 1. Introduction

**Figure 1:**
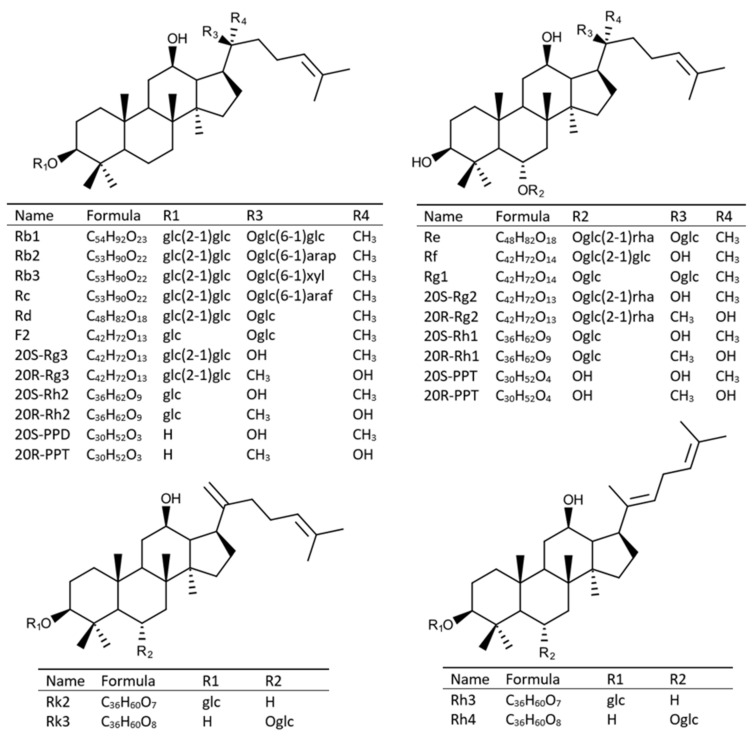
The chemical structures and molecular formulas of reference standards in this study. The glc, arap, xyl, araf, and rha refer to β-D-glucopyranosyl, α-L-arabinopyranosyl, β-D-xylopyranosyl, α-L-arabinofuranosyl, and α-L-rhamnopyranosyl, respectively.

*Panax ginseng* root has been used as a natural medicinal herb for thousands of years in Asian countries, especially in China, Korea, and Japan. It has become one of the most popular and best-selling herbs in the world herb market [1,2]. Ginseng saponins, known as ginsenosides, are thought to be the main bioactive components of ginseng and they have wide health-promoting effects, including anti-ageing [3], anti-stress [4], antioxidative [5], antifatigue [6], anti-diabetes [7], anticancer [8], enhanced liver function [9], improved immune system [10], and improved climacteric disorder and sexual function [11]. To date, nearly two hundred ginsenosides have been isolated and identified from various tissues of ginseng plants [12]. Based on the chemical structures of aglycones moieties, ginsenosides are mainly divided into protopanaxadiol (PPD) type ginsenosides, such as Rb1, Rb2, Rc, Rd, Rg3, and so on, or protopanaxatriol (PPT) type ginsenosides, for example, Rg1, Re, Rg2, and Rh1, and so on (Figure 1).

Traditionally, ginseng root has been regarded as the most used and valuable part for health care and medical applications. Thus, ‘ginseng’ in literature and media refers to ginseng root unless otherwise specified. Ginseng harvested from the wild or farm is fresh ginseng. Fresh ginseng easily decomposes, because of the high water content (approximately 70–80%), and might coexist with soil microorganisms [13], thus it is rarely used. For long-term storage and improved pharmacological effects, fresh ginseng is usually processed before application and usage, mainly through heat-involved processes, such as steaming [14], toasting [15], baking [16], and fermentation [17]. The common commercially processed ginseng products are white ginseng (WG), red ginseng (RG), and black ginseng (BG). WG is produced by dehydrating fresh ginseng in sunlight; RG is produced by steaming fresh ginseng at 95–100 °C for 2–3 h; and, BG is produced through nine cycles of steaming fresh ginseng at 95–98 °C for 3 h [18,19]. WG mainly contains ginsenosides m-Rb1, m-Rb2, m-Rc, Re, and Rg1. The main ginsenosides of RG are Rb1, Rb2, Rc, Rd, Re, and Rg1 [20], while BG contains some unique ginsenosides, such as Rd, Rk1, Rg5, and Rg3, which are not present in WG, and exhibit more potent pharmacological effects than WG and RG [21,22,23]. The less polar ginsenosides in black ginseng can be deglycosylated from major polar ginsenosides during steaming fresh ginseng. Among the less polar ginsenosides, Rg3 is regarded as the bioactive component that exerts pharmacological effects, especially the anti-diabetic effect [24]. Increasing the content of ginsenoside Rg3 through post-harvest processing is considered to be an effective strategy for improving the pharmacological function of ginseng.

Phytochemical studies have found that the ginseng leaf contains abundant ginsenosides, and the total content of ginsenosides in the leaf is higher than in the root [25,26,27]. Unlike ginseng root, ginsenosides Re and Rd are the major ginsenosides in the ginseng leaf [26,27]. Moreover, ginseng leaf extract has many pharmacological activities that are similar to ginseng root extract [28]. Thus, ginseng leaves have advantages over ginseng root in terms of cost, source availability, and sustainability, for ginseng leaves can be harvested annually while ginseng root usually takes four to six years to be harvested. At a minimum, ginseng leaf can be used as a valuable source of ginsenosides Re and Rd [29,30].

Steaming ginseng leaves can produce some new ginsenosides and enhance the leaves’ potential. Five new dammarane ginsenosides (SL1-SL3, ST1, and ST2) were isolated from steamed ginseng leaves [31,32]. Extracts from steamed ginseng leaves have been found to enhance cytotoxic effects on human leukemia HL-60 cells [32]. Another study found ginsenosides that were extracted from steamed ginseng leaves and flowers inhibited LPS-induced IL-12 production [33]. However, there are no publications concerning changes in ginsenoside profiles during the leaf steaming process. Ginsenoside Rd can be converted into Rg3 when ginseng is treated with heat-processing [12]. The purpose of this study is to find out whether it is possible to increase the content of ginsenoside Rg3 in black ginseng leaves by following the same process used to make black ginseng root. This study investigated the changes of ginsenosides during steaming ginseng leaves to provide chemical information for the development and application of ginseng leaf.

## 2. Results and Discussion

### 2.1. The Color Changes of Ginseng Leaves

**Figure 2:**
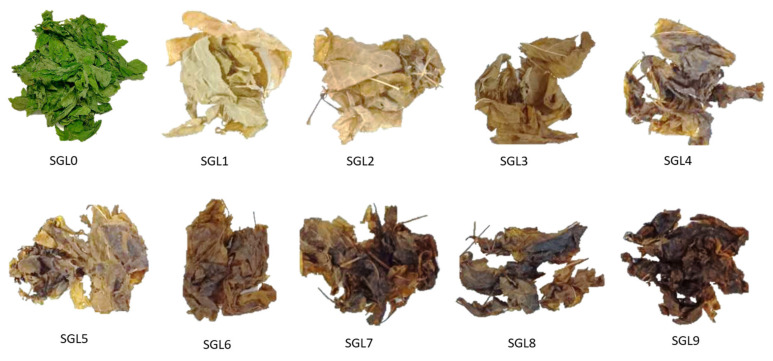
Pictures of steamed ginseng leaves (SGL) at different steaming cycles.

From Figure 2, we can see that the color of the ginseng leaves gradually became darker with the increased steaming cycles. The leaf color was measured according to the color parameters L, a, and b, which are used by the International Commission on Illumination, in order quantify the changes of color during the steaming process. The L value corresponds to the lightness of color, with values ranging from 0 (black) to 100 (white). The a and b values are color coordinates expressed numerically, where a represents red or green color and b represents the yellow or blue color.

Table 1 shows that the L and b values of ginseng leaves decreased while the a value increased during the steaming process. A decrease in L value indicates color darkening during the steaming process. A decrease in negative a value, then an increase in positive a value indicates a decrease in the greenness, and an increase in the redness of the leaves during steaming. A decreased positive b value indicates a decrease in the yellowness. Therefore, the color measurements indicate some loss or change of color in ginseng leave during steaming, which provides an explanation for why it is called black ginseng or black ginseng leaf.

### 2.2. The Contents of Ginsenosides During Different Steaming Times

**Figure 3:**
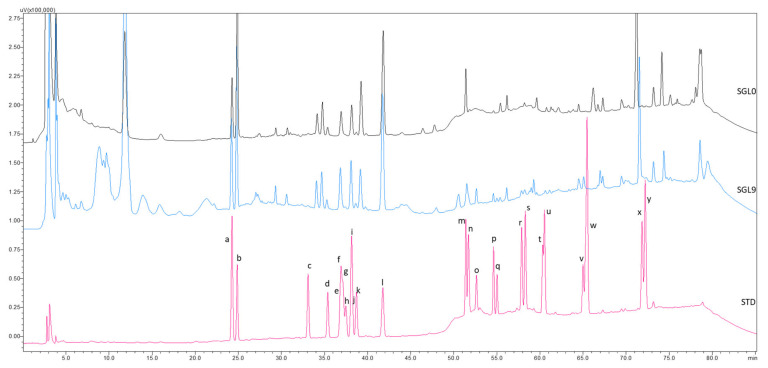
Off-set HPLC chromatograms of ginsenosides detected from the steamed ginseng leaves (SGL) as compared with the chromatogram of the ginsenoside standards. a, Rg1; b, Re; c, Rf; d, Rb1; e, 20S-Rg2; f, Rc; g, 20S-Rh1; h, 20R-Rg2; i, Rb2; j, 20R-Rh1; k, Rb3; l, Rd; m, F2; n, Rk3; o, Rh4; p, 20S-Rg3; q, 20R-Rg3; r, 20S-PPT; s, 20R-PPT; t, 20S-Rh2; u, 20R-Rh2; v, Rk2; w, Rh3; x, 20S-PPD; y, 20R-PPD.

The ginsenosides extracted from steamed ginseng leaves were well separated within 80 min. by the RP-C18 column. From Figure 3, we can see that some paired isomers of less polar ginsenosides were completely separated, such as Rk3 and Rh4, 20S-Rg3, and 20R-Rg3, 20S-PPT and 20R-PPT, 20S-Rh2, and 20R-Rh2, Rk2, and Rh3, and 20S-PPD and 20R-PPD. The peaks of the polar ginsenosides did not change much, but the peaks of the less polar ginsenosides became more pronounced with each steam cycle (Figure S1), especially ginsenoside Rg3. There are relatively big peaks of Rg3 in the SGL9 sample, as compared to no Rg3 peaks in the SGL0 sample.

Sixteen ginsenoside references were used to conduct the quantitative analysis on the ginsenosides changes during the leaf steaming process. The calibration curves were plotted based on the linear regression of the integrated peak areas (y) to concentrations (x, mg) of 16 ginsenoside references in the standard solution at five different concentrations. Table S1 shows the regression equations of calibration curves, correlation coefficient, and linear ranges for the ginsenoside standards. The validations of intraday and interday precisions are from 0.06% to 4.90% and from 3.38% to 6.50%, respectively (Table S2). A recovery experiment was carried out to evaluate the accuracy of the method, and the recoveries of 16 ginsenosides were between 91.25% and 106.11%, which are listed in Table S3.

**Figure 4:**
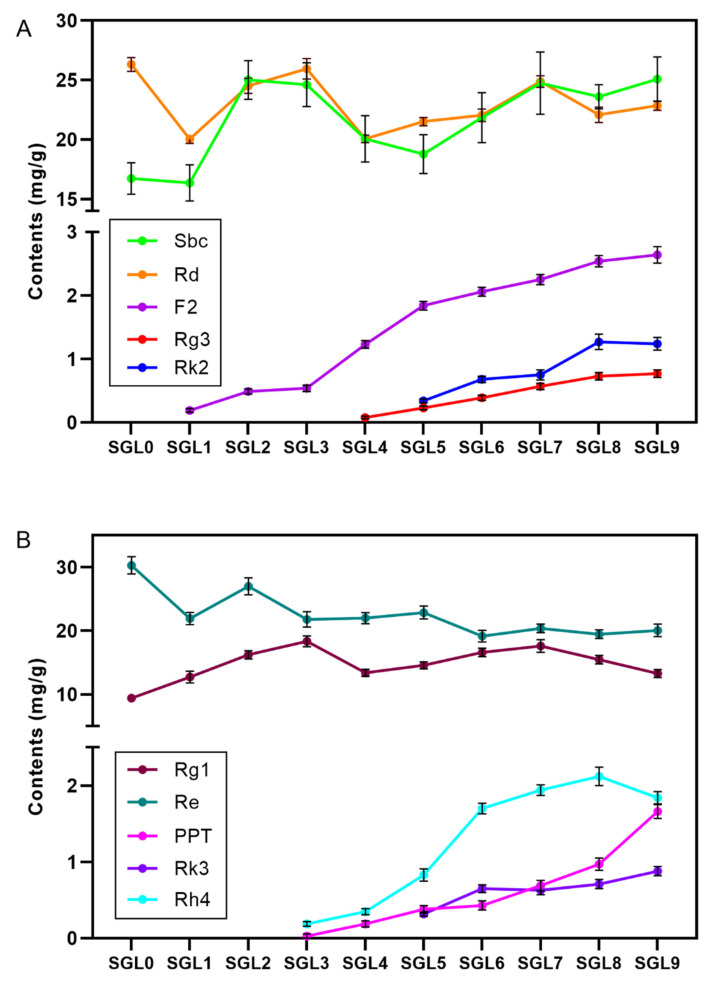
The contents of protopanaxadiol (PPD)-type ginsenosides (**A**) and PPT-type ginsenosides (**B**) during different steaming batches. Sbc refers to the sum of ginsenosides Rb1, Rb2, Rb3, and Rc. Rg3 refers to the 20S-Rg3 and 20R-Rg3.

The contents of 16 ginsenosides from different batches of steamed ginseng leaves were quantified (Table S4). The amount of less polar ginsenosides (F2, Rg3, Rk2, Rk3, Rh3, Rh4, and PPT), which were not present in the raw ginseng leaf, accounted for 10.4% of the quantified ginsenosides after steamed nine times. From Figure 4, we can see the changes of ginsenoside content in different steamed cycles. Overall, although the contents of the polar PPD ginsenosides fluctuated in different steamed batches, the content of Rd tended to decrease from 26.32 mg/g at the beginning to 22.85 mg/g in the SGL9, while the total content of ginsenoside Rb1, Rb2, Rb3, and Rc (these four ginsenosides were marked Sbc) showed an increasing trend with steaming. The less polar ginsenosides could not be quantified in the raw ginseng leaves, and ginsenosides F2, Rg3, and Rk2 appeared from SGL1, SGL4, and SGL5, respectively. After being produced, the contents of the less polar ginsenosides F2, Rg3, and Rk2 increased by 12.9 folds, 8.6 folds, and 2.6 folds, respectively, at the ninth steamed leaves (Figure 4A). The content change of PPT-type ginsenosides was similar to that of the PPD-type ginsenosides with steaming (Figure 4B). The content of ginsenoside Re gradually decreased by 33.8% after being steamed nine times and ginsenoside Rg1 slightly increased with steaming. The less polar ginsenosides PPT and Rh4 appeared from SGL3 and accumulated relatively rapidly, and Rk3 emerged from SGL5 and increased weakly.

**Figure 5:**
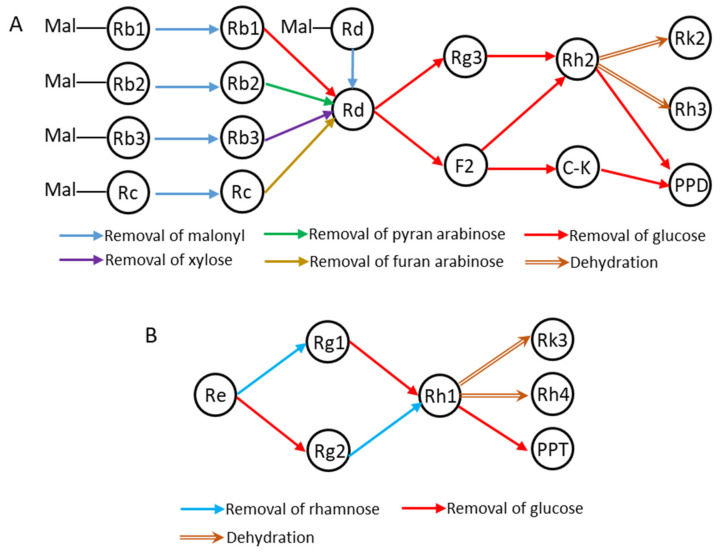
The transformation pathway of PPD-type ginsenosides (**A**) and protopanaxatriol (PPT)-type ginsenosides (**B**) during the steaming process.

From the above results, we found the less polar ginsenosides (both PPD-type and PPT-type), which were not present in the raw ginseng leaves, produced and accumulated through repeated steaming. The literature reported that the less polar ginsenosides can be transformed from the polar ginsenosides with heat process [12]. Briefly, the ginsenosides with big molecules, such as Rb1, Rb2, Rb3, and Rc, can transform to Rd by losing one sugar residue, and then ginsenoside Rd continues to lose sugars to from less polar ginsenosides. This would explain why the contents of the less polar PPD-type ginsenosides (F2, Rg3, and Rk2) increased as the amount of ginsenoside Rd decreased with steaming. If ginsenoside Rd is sourced from ginsenosides Sbc with heat process, the amount of Sbc should decrease with steaming. However, the amount of Sbc fluctuated and showed the opposite trend with steaming. It suggests that other compounds can transform into Sbc, leading to an increasing trend of Sbc in the heat process. While, in our previous study we found that there was an abundance of malonyl ginsenosides, especially mal-Rb2, mal-Rc and mal-Rd, in ginseng leaf [27]. The malonyl ginsenosides are thermally unstable and they can be transformed into their neutral ginsenosides by losing the malonyl group [34]. Literature reported that the contents of malonyl ginsenosides (mal-Rb1, mal-Rb2, mal-Rb3, mal-Rc, and mal-Rd) decreased in the steamed ginseng and red ginseng when compared to the fresh ginseng, accordingly, their neutral ginsenosides enriched in the heat-treated ginseng [13]. Unsurprisingly, the malonyl ginsenosides would lose the malonyl group to produce ginsenosides Sbc and Rd and then lost some sugar groups to form the less polar PPD type ginsenosides during the steaming process of ginseng leaf. Apart from the demalonylation and deglycosylation, the less polar ginsenoside Rk2 and Rh3 can be produced from ginsenoside Rh2 by losing an H_2_O to form a double bond between C-20 and C-21, and C-20 and C-22, respectively. The transformation pathway of PPD-type ginsenosides during the steaming process are displayed in Figure 5A based on our results and literature. Similarly, Figure 5B presents the relationship between PPT-type ginsenosides. Because there were little malonyl PPT-type ginsenosides in the raw ginseng leaf [27], ginsenoside Re degraded to small ginsenosides during the steaming, but cannot be supplied from other compounds, leading to gradually decrease with steaming. Thus, steaming ginseng leaf can produce ginsenoside transformation similar to that of black ginseng.

In the process of making black ginseng, Lee et al found that the less polar ginsenosides can be detected after steaming once and their amount increased with the number of steaming times and peaked in red ginseng steamed eight times [14]. While with our ginseng leaf, the less polar ginsenosides (except for F2) cannot be detected until steaming three times, some can be detected from four times or even from nine times. This is probably due to the short steaming time. In the preparation of black ginseng, the steaming time is about 2 or 3 h each cycle [14,35], while our ginseng leaf was steamed for 30 min. each cycle, when considering that ginseng leaves are much thinner than ginseng root. This might be the same reason why the polar ginsenoside content in our steamed leaves is much higher than the less polar ginsenoside content after nine cycles of steaming treatment. It suggests that ginseng leaf needs to be steamed longer time each cycle (greater than 30 min.) to produce black ginseng leaf. Another interesting thing we found that PPT-type ginsenosides losing sugars to yield the aglycones PPT seems easier than that PPD-type ginsenosides degrading to the aglycones PPD. Ginsenoside PPT was detected from SGL3 and its amount continued to increase to 1.66 mg/g at SGL9, while the aglycones PPD cannot be detected during the whole steaming treatment. In black ginseng root, the content of aglycones PPT was found 20 times more abundant than that of aglycones PPD (10.96 mg/g PPT vs 0.52 mg/g PPD) [18]. In addition, several studies found there were abundant of ginsenosides Rk1 and Rg5 [14,18,20,22,36,37] and relatively content of ginsenosides Rk2 and Rh3 [20,37] in the black ginseng or steamed ginseng. The polar PPD-type ginsenosides (Rb1, Rb2, Rc, Rd, etc.) are more likely to be converted to ginsenosides Rk1 and Rg5 by dehydration from Rg3 and to ginsenosides Rk2 and Rh3 through losing an H_2_O from Rh2 than to be completely degraded to the aglycones PPD during heat process.

In the application of ginseng or ginseng products, residual pesticide is not only a safety issue, but also reduces the value of medicinal effect. Most ginseng is commercially planted at a high density under artificial shade nets in the field [38]. Pesticides (spray on ginseng leaves) are frequently applied to control the various fungal diseases that affect ginseng growing at high planting densities [38]. Pesticides can remain in the ginseng plant. In particular, the amount of pesticide residues in ginseng leaves far exceed residues in ginseng roots [39,40], which greatly affects the consumption of ginseng leaves. This might be the reason why ginseng leaf is not widely used. However, the ginseng leaves used in this study were from New Zealand forest grown ginseng, which were grown under pine forest in an open wild environment as our previous reports [27,41,42]. No pesticides were used during the growing stage of ginseng. That means that New Zealand forest grown ginseng leaves can be widely used to produce ginsenosides without pesticide residues. The steaming process of New Zealand forest grown ginseng leaf will provide a source of less polar ginsenosides. As the first report of trying to produce black ginseng leaf, this proof-of concept study used relatively short steaming times at each cycle since the used leaves are thin. We suggest that the steaming time could be appropriately extended to yield more less polar ginsenosides when the ginseng leaves are used as raw material to manufacture less polar ginsenosides.

## 3. Materials and Methods

### 3.1. Ginsenoside Standards, Chemicals and Reagents

Twenty-five reference standards of ginsenosides (structures are shown in Figure 1) (Rb1, Rb2, Rb3, Rc, Rd, Re, Rf, Rg1, 20S-Rg2, 20R-Rg2, 20S-Rg3, 20R-Rg3, 20S-Rh1, 20R-Rh1, 20S-Rh2, 20R-Rh2, Rh3, Rh4, Rk2, Rk3, F2, 20S-PPD, 20R-PPD, 20S-PPT, and 20R-PPT) were purchased from Star Ocean Ginseng Ltd (Suzhou, Jiangsu, China). The purities of all reference standards were no less than 98.0%. HPLC-grade methanol (MeOH) and acetonitrile (MeCN) were purchased from Fisher Chemical (Pittsburg, PA, USA). Water (deionized) was obtained from a Milli-Q Ultra-pure water system (Millipore, Billerica, MA, USA). The other reagents used in this study were of analytical grade.

### 3.2. Ginseng Leaf Preparation

Fresh ginseng leaves (*Panax ginseng*) were collected in May 2019 from pine forests around Taupo, New Zealand. Two hundred grams of fresh ginseng leaves were steamed in a household steamer at 98–99 °C for 30 min. After steaming, the leaves were dried in a drying oven at 60 °C for 1.5 h. This process was repeated nine times. The color parameters were measured with a CR-400 Chroma Meter (Konica Minolta, Ramsey, NJ, USA). Each sample (Figure 2) was ultrasonically extracted three times using a Q700 sonicator (Qsonica, Melville, NY, USA), as our previous methods [27,42]. Briefly, 0.7 g dried steamed ginseng leaf pieces were incubated with 10 mL 70% (*v/v*) aqueous MeOH for 30 min. and then extracted at 20 kHz for 10 min at no more than 40 °C (10 min. extraction contained five cycles, each cycle consisted of 2 min. ultrasonic extraction at 15% amplitude and 1 min. for cooling between extraction). The supernatant was collected after centrifugation (4000 rpm for 10 min) and the sediment was extracted twice more. The three extracts were mixed and filtered through a 17 mm (0.2 μm) filter before HPLC injection.

### 3.3. Ginsenoside Reference Standards Preparation

Twenty-five reference standards of ginsenosides (Rb1 (0.769 mg/mL), Rb2 (0.846 mg/mL), Rb3 (0.629 mg/mL), Rc (1.077 mg/mL), Rd (0.692 mg/mL), Re (0.923 mg/mL), Rf (1.462 mg/mL), Rg1 (1.154 mg/mL), 20S-Rg2 (0.615 mg/mL), 20R-Rg2 (0.846 mg/mL), 20S-Rg3 (1.154 mg/mL), 20R-Rg3 (0.769 mg/mL), 20S-Rh1 (1.000 mg/mL), 20R-Rh1 (0.769 mg/mL), 20S-Rh2 (1.077 mg/mL), 20R-Rh2(1.231 mg/mL), Rh3 (0.769 mg/mL), Rh4 (0.923 mg/mL), Rk2 (0.692 mg/mL), Rk3 (0.923 mg/mL), F2 (0.692 mg/mL), 20S-PPD (0.769 mg/mL), 20R-PPD (1.077 mg/mL), 20S-PPT (1.231 mg/mL), and 20R-PPT (0.769 mg/mL)) were dissolved in 70% MeOH and then mixed and diluted with 70% MeOH in order to obtain a series of mixture standard solutions of different concentrations. The solutions were filtered through a 17 mm (0.2 μm) syringe filter before HPLC injection.

### 3.4. HPLC Analysis

The HPLC instrument used in this study was a Shimadzu prominence LC-20A UFLC stack HPLC system (Shimadzu, Kyoto, Japan) equipped with a DGU-20A3 degasser, LC-20AD pump, CTO column oven, SPD-20A detector, and SIL-20A autosampler. A Waters Symmetry Shield RP18 column (4.6 mm × 250 mm, 5 μm, 100 Å) was used. The mobile phase consisted of deionized water (A) and acetonitrile (B). The gradient elution program was as follows: 0–15 min., 20% B; 15–25 min., 20–31% B; 25–35 min., 31–35% B; 35–40 min., 35–36% B; 40–45 min., 36–39% B; 45–75 min., 39–95%; 75–85 min., 95–20% B, and 85–90 min., 20% B. The column temperature was set at 25 °C and the flow rate was 1 mL/min. The sample injection volume was 20 μL and the wavelength of detection was set at 203 nm.

## 4. Conclusions

This paper provides the ginsenoside changes during nine cycles’ steaming ginseng leaf (black ginseng leaf) for the first time. The major ginsenosides in the raw ginseng leaf converted into the less polar ginsenosides after nine cycles of steaming New Zealand forest grown ginseng leaves. It suggests that ginseng leaf can be used as a source to manufacture the less polar ginsenosides by this heat process.

## Figures and Tables

**Table 1:** Changes in color parameters of ginseng leaves during the steaming at different steaming cycles.

Sample	SGL0	SGL1	SGL2	SGL3	SGL4	SGL5	SGL6	SGL7	SGL8	SGL9
Time (min)	0	30	60	90	120	150	180	210	240	270
L	53.50 ± 5.09	44.81 ± 1.05	40.03 ± 4.47	37.11 ± 3.72	32.29 ± 3.66	33.59 ± 7.06	31.33 ± 1.83	28.70 ± 3.35	28.78 ± 3.19	28.02 ± 1.92
+a	−26.80 ± 3.83	−2.72 ± 1.13	−0.62 ± 0.11	0.97 ± 0.52	1.15 ± 0.34	1.51 ± 0.39	1.66 ± 0.55	2.10 ± 0.53	1.62 ± 0.33	1.73 ± 0.10
+b	44.83 ± 6.79	15.20 ± 2.94	13.83 ± 2.32	13.18 ± 2.60	8.58 ± 2.08	11.50 ± 5.18	7.04 ± 2.13	6.65 ± 2.18	5.08 ± 4.87	2.50 ± 1.34

L indicates lightness, +a is the red coordinate, and +b is the yellow coordinate.

## Supplementary Materials

The following are available online, Figure S1: Off-set HPLC chromatograms of ginseng leaves at different steaming cycles, Table S1: The regression equations, linear ranges, limits of detection and limits of quantification of ginsenoside standards, Table S2: Inter-day variation and intra-day variation (RSD) of the ginsenoside standards. Table S3: The recovery of ginsenoside standards. Table S4: The sixteen ginsenoside contents (mg/g) in the ginseng leaves with different steamed cycles.
