# Supplementary material for: Changes of Ginsenoside Composition in the Creation of Black Ginseng Leaf
Source: Molecules. 2020 Jun 18;25(12):2809. doi: 10.3390/molecules25122809 (PMC7355439; doi:10.3390/molecules25122809)
Supplement: Supplementary file 1 [file molecules-25-02809-s001.pdf]

Supplementary data

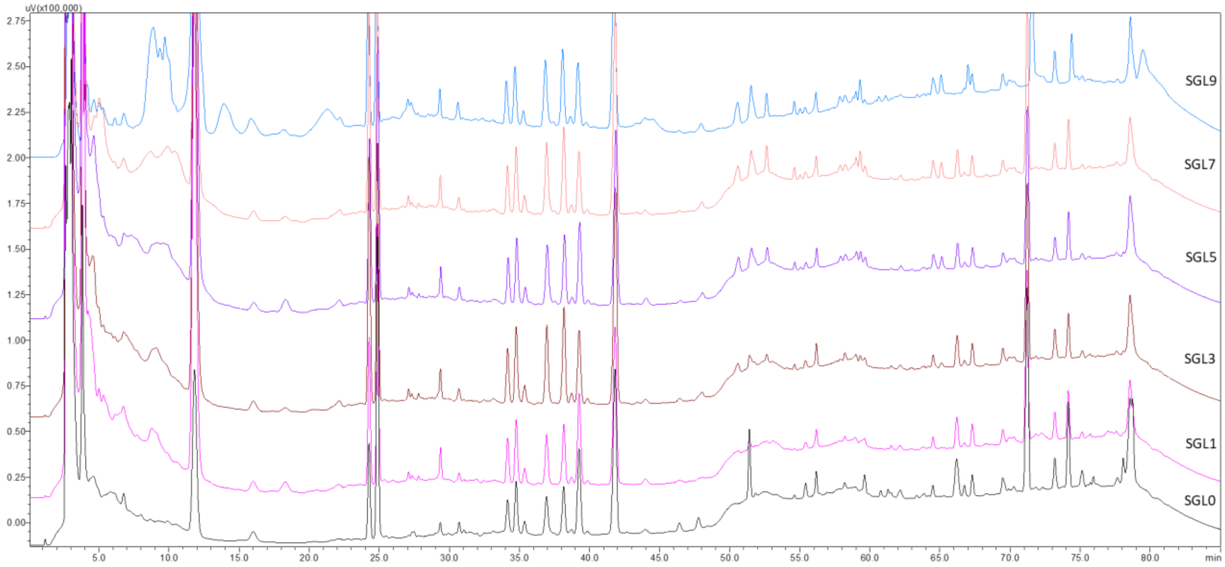

Figure S1. Off-set HPLC chromatograms of ginseng leaves at different steaming cycles.

Table S1. The regression equations, linear ranges, limits of detection and limits of quantification of ginsenoside standards

Ginsenosides	Calibration curve	r^2	Concentration range (mg/L)	LOD (mg/L)	LOQ (mg/L)
Rg1	y=6650.9x+20748	0.9999	20.59-1029.41	13.35	44.51
Re	y=6049.6x+21738	0.9999	12.35-617.65	6.35	21.15
Rf	y=7658.4x+3491	0.9996	10.35-517.65	2.51	8.38
Rb1	y=6022.2x+2221	0.9999	9.53-476.47	1.79	5.97
Rg2	y=8133.7x+52824	0.9997	8.48-481.84	1.03	3.44
Rh1	y=7597.5x+10594	0.9999	9.18-458.82	6.20	20.67
Rc	y=5720.8x-2278	0.9995	11.29-564.71	1.38	4.61
Rb2	y=5228.0x+2227	0.9994	11.88-594.18	2.10	7.01
Rb3	y=5480.0x+2626	0.9998	11.06-552.94	2.38	7.92
Rd	y=6204.5x+2683	0.9998	12.94-647.06	2.47	8.23
F2	y=6957.8x+23124	0.9995	2.40-485.44	1.71	5.64
Rg3	y=8331.3x+28160	0.9998	7.65-382.35	1.81	6.04
20R-Rg3	y=5313.9x+6110.3	0.9981	8.35-323.53	2.20	7.33
Rk3	y=5950.8x+18810	0.9993	5.53-386.23	1.87	6.23
Rh4	y=5656.1x+27710	0.9989	4.71-412.34	2.96	9.86
Rk2	y=4676.8x+70784	0.9991	6.31-389.26	4.67	15.55
Rh3	y=4909.8x+45090	0.9994	5.76436.85	2.38	7.93
Rh2	y=12634x+1084	0.9999	6.00-300.00	0.98	3.25
PPT	y=9181.3x+6620	0.9998	8.18-405.88	2.57	8.56
PPD	y=14733x+11324	0.9999	6.71-335.29	3.35	11.18

y is the integrated peak area and x is the concentration of analyte (mg/L). LOD, limit of detection; LOQ: limit of quantification.

Table S2. Inter-day variation and intra-day variation (RSD) of the ginsenoside standards. Intra-day and inter-day variations were used to evaluate the precision of this HPLC method for determining ginsenoside. Ginsenoside standards with the known concentrations were mixed together and tested. In the intra-day variability experiment, the test was conducted within one day and three parallel experiments were carried out. While for the inter-day precision, the mixed standard solutions were examined in three different days. The relative standard deviation was used to describe variations.

Name	Inter-day		Intra-day	
	Con.(mg/L)	RSD	Con.(mg/L)	RSD
Rg1	134.68	5.46%	133.98	1.87%
Re	76.21	4.26%	77.18	2.31%
Rf	63.78	6.31%	64.87	1.22%
Rb1	59.56	2.58%	59.65	0.06%
Rg2	51.35	5.63%	51.96	2.67%
Rh1	55.32	4.75%	54.49	1.36%
Rc	69.24	4.12%	70.25	0.67%
Rb2	74.73	2.57%	75.31	1.34%
Rb3	69.84	5.26%	68.59	0.36%
Rd	80.78	6.06%	81.36	2.37%
F2	56.67	4.68%	57.05	3.61%
Rg3	46.79	5.34%	47.82	4.37%
20R-Rg3	40.45	6.50%	41.34	4.90%
Rk3	76.92	3.62%	77.62	1.30%
Rh4	72.34	4.37%	73.31	2.51%
Rk2	57.64	5.73%	57.99	1.37%
Rh3	64.08	4.19%	64.93	2.57%
Rh2	68.73	4.73%	68.98	1.13%
PPT	49.69	3.64%	50.23	2.34%
PPD	41.72	3.91%	41.96	1.79%

Table S3. The recovery of ginsenoside standards. The recoveries were expressed according to the following formula: recovery (%) = (found amount original amount)/added amount X100. Ginsenoside standards with known amount were added into a certain amount (0.5 g) of the sample.

Ginsenosides	Original (μg)	Added (μg)	Found (μg)	Recovery (%)	RSD (%)
Rg1	222.61	10.96	232.63±1.12	91.42	10.22
Re	169.57	10.61	179.26±1.01	91.33	9.52
Rf	69.25	4.39	73.77±0.28	102.96	6.38
Rb1	211.13	16.53	226.81±1.34	94.88	8.11
Rg2	15.29	1.54	16.93±0.17	106.49	11.04
Rh1	2.73	1.00	3.77±0.03	104.00	3.00
Rc	75.03	5.39	80.25±0.22	96.85	4.08
Rb2	72.07	6.35	78.38±0.11	99.37	1.73
Rb3	19.78	1.73	21.61±0.06	105.78	3.47
Rd	41.12	2.60	43.73±0.21	100.38	8.08
F2	0.09	1.38	1.56±0.02	106.52	1.45
Rg3	0.72	1.15	1.96±0.03	107.83	2.61
20R-Rg3	0.00	1.28	1.17±0.05	91.41	3.91
Rk3	0.00	0.96	0.93±0.04	96.88	4.17
Rh4	0.00	0.88	0.82±0.07	93.18	7.95
Rk2	0.00	1.26	1.28±0.07	101.59	5.56
Rh3	0.00	0.96	0.94±0.06	97.92	6.25
Rh2	0.12	1.35	1.36±0.01	92.00	1.05
PPT	0.00	1.34	1.30±0.04	97.01	2.99
PPD	0.00	1.19	1.25±0.07	105.04	5.88

Table S4. The sixteen ginsenoside contents (mg/g) in the ginseng leaves with different steamed cycles.

Ginsenosides	SGL0	SGL1	SGL2	SGL3	SGL4	SGL5	SGL6	SGL7	SGL8	SGL9
Rg1	9.41±0.12	12.74±0.23	16.24±0.14	18.35±0.24	13.40±0.23	14.59±0.17	16.61±0.16	17.62±0.27	15.47±0.26	13.30±0.11
Re	30.30±0.45	21.95±0.34	27.00±0.36	21.80±0.24	22.00±0.28	22.88±0.24	19.16±0.19	20.40±0.19	19.48±0.21	20.06±0.27
Rb1	1.39±0.03	1.36±0.02	2.99±0.07	1.42±0.06	1.45±0.04	1.37±0.05	1.37±0.06	1.52±0.04	1.46±0.03	1.27±0.02
Rc	6.95±0.11	6.51±0.09	9.24±0.07	9.80±0.03	8.17±0.07	7.72±0.17	9.13±0.16	10.33±0.11	10.30±0.21	10.86±0.24
Rb2	7.95±0.19	7.92±0.17	11.58±0.32	12.25±0.23	9.25±0.24	8.72±0.31	10.04±0.36	11.37±0.34	10.50±0.14	11.09±0.19
Rb3	0.43±0.01	0.56±0.01	1.21±0.02	1.15±0.03	1.18±0.01	0.97±0.02	1.32±0.03	1.53±0.05	1.35±0.04	1.87±0.03
Rd	26.32±0.58	19.22±0.34	24.52±0.63	25.95±0.87	20.06±0.32	21.51±0.34	22.04±0.52	24.88±0.48	22.08±0.64	22.85±0.39
F2	#	0.19±0.02	0.49±0.03	0.54±0.01	1.23±0.03	1.84±0.01	2.06±0.02	2.25±0.03	2.54±0.03	2.64±0.13
20S-Rg3	#	#	#	#	0.02±0.01	0.12±0.01	0.22±0.01	0.34±0.02	0.41±0.02	0.42±0.01
20R-Rg3	#	#	#	#	0.06±0.03	0.11±0.02	0.17±0.03	0.23±0.01	0.31±0.03	0.36±0.05
PPT	#	#	#	0.03±0.01	0.19±0.03	0.38±0.05	0.43±0.02	0.69±0.01	0.97±0.03	1.66±0.03
Rk3	#	#	#	#	#	0.32±0.02	0.65±0.02	0.63±0.06	0.71±0.04	0.88±0.06
Rh4	#	#	#	0.19±0.01	0.35±0.01	0.83±0.08	1.70±0.03	1.94±0.04	2.12±0.12	1.84±0.08
Rk2	#	#	#	#	#	0.34±0.03	0.68±0.03	0.75±0.02	1.27±0.12	1.24±0.04
Rh3	#	#	#	#	#	#	#	#	#	0.40±0.04
PPD	#	#	#	#	#	#	#	#	#	#
Sbc	16.72±1.32	16.35±1.52	25.02±1.63	24.62±1.85	20.05±1.95	18.78±1.63	21.85±2.10	24.75±2.62	23.60±1.02	25.09±1.86
Rg3	#	#	#	#	0.08±0.02	0.23±0.03	0.39±0.04	0.57±0.02	0.72±0.03	0.78±0.02

# refers to not quantifiable. Sbc refers to the sum of ginsenosides Rb1, Rb2, Rb3, and Rc. Rg3 refers to the 20S-Rg3 and 20R-Rg3.
